# Malignant T cells activate endothelial cells via IL-17 F

**DOI:** 10.1038/bcj.2017.64

**Published:** 2017-07-21

**Authors:** B Lauenborg, I V Litvinov, Y Zhou, A Willerslev-Olsen, C M Bonefeld, C Nastasi, S Fredholm, L M Lindahl, D Sasseville, C Geisler, M M Wasik, T Krejsgaard, L M R Gjerdrum, L Iversen, N Odum, A Woetmann

**Affiliations:** 1 Department of Immunology and Microbiology, University of Copenhagen, Copenhagen, Denmark; 2 Division of Dermatology, Ottawa Hospital Research Institute, University of Ottawa, Ottawa, ON, Canada; 3 Department of Dermatology and Skin Science, University of British Columbia, Vancouver, BC, Canada; 4 Department of Dermatology, Aarhus University Hospital, Aarhus, Denmark; 5 Division of Dermatology, McGill University Health Centre, Montréal, QC, Canada; 6 Department of Pathology and Laboratory Medicine, University of Pennsylvania, Philadelphia, PA, USA; 7 Department of Pathology, Zealand University Hospital, Roskilde, Denmark

Sir,

Cutaneous T-cell lymphoma (CTCL) defines a group of lymphoproliferative disorders that primarily affects the skin. Mycosis fungoides (MF) is the most common clinical variant of CTCL comprising almost 50% of all patients diagnosed with primary T-cell lymphoma.^1^ The etiology and pathogenesis of the disease remain poorly understood. Recent data suggest that environmental factors such as bacteria rather than heredity play an important role in the pathogenesis and disease progression.^2,3,4^ Although angiogenesis generally plays a key role in tumor growth and metastasis and is considered one of the hallmarks of cancer,^5^ little is known about angiogenesis in CTCL. However, recent data demonstrated that micro-vessel formation and the density of micro-vessels in CTCL skin lesions correlate with disease progression suggesting that angiogenesis plays a role in the pathogenesis.^6^ Notably, malignant inflammation defined as a pro-tumorigenic inflammatory environment orchestrated by the tumor cells plays a key role in disease progression (reviewed in ref. 7). Accordingly, we hypothesized that malignant T cells also orchestra angiogenesis in affected skin. In support, malignant T cells spontaneously produce angiogenic factors such as VEGF-A, VEGF-C and lymphotoxin-alpha^8, 9, 10, 11^ and induce increased vascularization and production of angiogenic factors *in vivo*.^12^ Recent observations indicate that IL-17 family cytokines stimulate and modulate oncogenic angiogenesis.^13^ Interestingly, due to abnormal activation of the JAK3/STAT3 signaling pathway, malignant T cells spontaneously express IL-17F and a fraction of MF patients display increased levels of IL-17A and/or IL-17F in lesional skin at levels comparable to those found in skin lesions from patients with psoriasis,^14^ a disease which is also associated with increased angiogenesis.^15^ Since malignant MF T cells constitutively express IL-17F and the expression in lesional skin is associated with progressive disease,^14^ we hypothesized that malignant T cells partly promote angiogenesis through the expression of IL-17F in MF patients. Accordingly, we plated endothelial cells (HUVEC) on growth factor depleted matrigel *in vitro* with or without culture supernatant from malignant T cell lines (MyLa2059 and PB2B cells both of which spontaneously produce IL-17F^14^) to assay for IL-17F mediated induction of endothelial sprouting and tube formation as described elsewhere.^11^

As shown in Figure 1, supernatant from the malignant T cell line MyLa2059 rapidly induced strong sprouting and tube formation (Figure 1b versus Figure 1a). Importantly, an IL-17F neutralizing antibody inhibited the endothelial response (Figure 1c versus Figure 1b) whereas an anti-IL-17A antibody (as a control) did not (Figure 1d versus Figure 1c) which is in agreement with the observation that MyLa2059 did not express IL-17A.^14^ The effect of IL-17F neutralization on the endothelial response was comparable to VEGF-A neutralization (Supplementary Figures S1A–S1E). Essentially similar responses were seen in a series of three independent experiments with MyLa2059 supernatants (Figure 2a) and in independent experiments using supernatants from another IL-17F producing malignant T cell line (the PB2B cell line) (Supplementary Figure S2, and data not shown). On average, endothelial responses to culture supernatants from malignant T cells were significantly inhibited by about 30% by the anti-IL-17F neutralizing antibody (Figure 2a, column 2 versus 4) whereas the control antibody as anticipated had no effect alone or in combination with anti-IL-17F antibody (Figure 2a) indicating that IL-17F produced by malignant T cells triggered endothelial activation as evidenced by an increased branching. In accordance, HUVEC cells expressed IL-17 receptor A (IL-17RA) and IL-17RC (data not shown) and exogenous recombinant IL-17F induced increased branching in endothelial cells confirming that IL-17F *per se* is able to activate endothelial cells (Figure 2b, column 4 and 5). Expectedly, recombinant IL-17A (Figure 2b, column 2 and 3) and the well-characterized angiogenic factor VEGF-A (Figure 2b, column 7) also induced enhanced endothelial cell branching and tube formation. Neutralization of autocrine VEGF-induced signaling did not affect STAT3 activation in malignant T cells (Supplementary Figure S3). We have previously shown that STAT3 signaling pathway drives malignant IL-17F expression.^14^ Together, our finding indicates that therapeutic inhibition of classic angiogenic pathways, like VEGF, will not affect IL-17F production by malignant T cells. As mentioned above, the malignant T cells in question did not produce IL-17A, but it is seems likely that IL-17A producing malignant T cells may also contribute to the induction of angiogenesis in CTCL patients. Interestingly, simultaneous expression of IL-17A and IL-17F by malignant T cells leads to IL-17A/IL-17F heterodimer formation in malignant supernatant^14^ and as shown in Figure 2b (column 6) IL-17A and IL-17F induce an enhanced response when compared to either cytokine alone. Some patients with CTCL display high levels of IL-17A, others display high levels of IL-17F, while some display high levels of both cytokines in their lesional skin.^14^ The average expression of IL-17A and IL-17F was increased in advanced stages of CTCL when compared to early stages, indicating that both IL-17 family cytokines may be involved in disease progression although only the correlation between IL-17F and progressive disease was statistically significant.^14^ Thus, it is conceivable that it is the total level of IL-17A/IL-17F (alone or in combination) that determines the impetus of these cytokines on the angiogenesis in MF.

Taken together, the present findings suggest that malignant T cells orchestra angiogenesis and malignant inflammation in tandem, which might play an important role during the accelerating disease progression observed in advanced stages of the disease.

In conclusion, the present study provides the first evidence that malignant T cells stimulate angiogenesis through release of IL-17F suggesting that IL-17F might serve as a novel target for anti-angiogenic therapy.

## Figures and Tables

**Figure 1:**
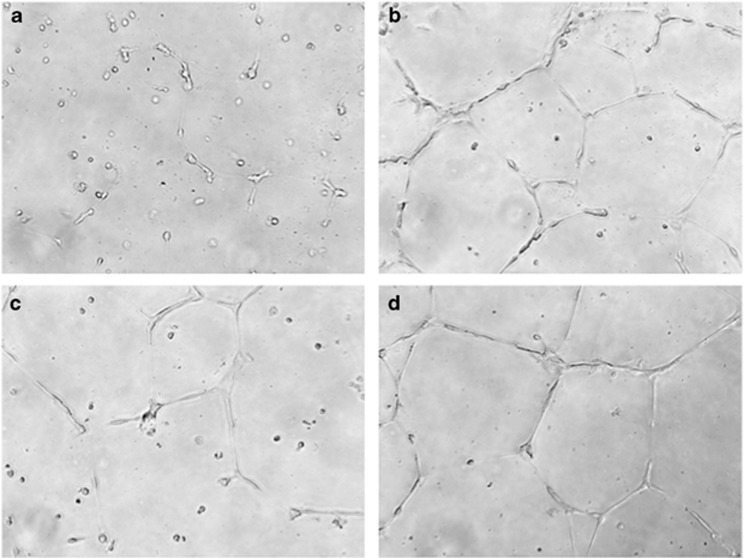
Malignant T cells (MyLa2059) trigger IL-17F- mediated endothelial tube formation. Endothelial tube formation assays were performed on growth factor reduced matrigel in 24-well plates. HUVEC cell sprouting when cultured with (**a**) M200 medium, (**b**) supernatant (10% vol/vol) from a malignant T cell line (MyLa2059), (**c**) MyLa2059 supernatant+anti-IL-17F antibody, and (**d**) MyLa2059 supernatant+anti-IL-17A antibody.

**Figure 2:**
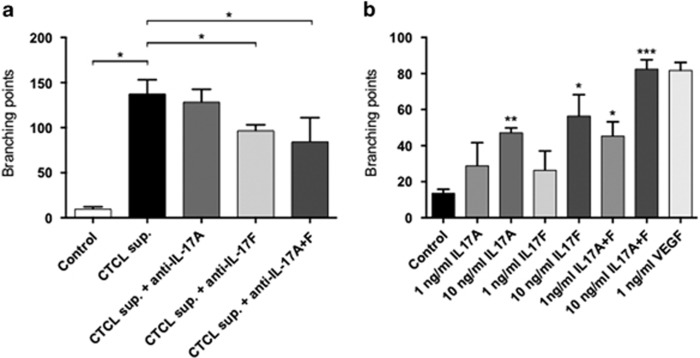
IL-17F increases the number of branching points and tube formation. Pictures of cultures were taken and the number of branching points counted representing the morphogenic activity of HUVEC cells following incubation with (**a**) malignant CTCL cell line (MyLa2059) supernatant (sup.) either alone or supplemented with anti-IL-17A or anti-IL-17F antibodies, *P<0.05 (paired *t*-test), or (**b**) in the presence of rhIL-17A, rhIL-17F, rhIL-17A+rhIL-17F or VEGF-A for 12  h. Bars represent mean values of three independent experiments. *P<0.05, **P<0.01, ***P<0.001 compared to control (paired *t*-test).
