# Supplementary material for: Malignant T cells activate endothelial cells via IL-17 F
Source: Blood Cancer J. 2017 Jul 21;7(7):e586–. doi: 10.1038/bcj.2017.64 (PMC5549256; doi:10.1038/bcj.2017.64)
Supplement: Supplementary Figures S1-S3 [file bcj201764x1.docx]

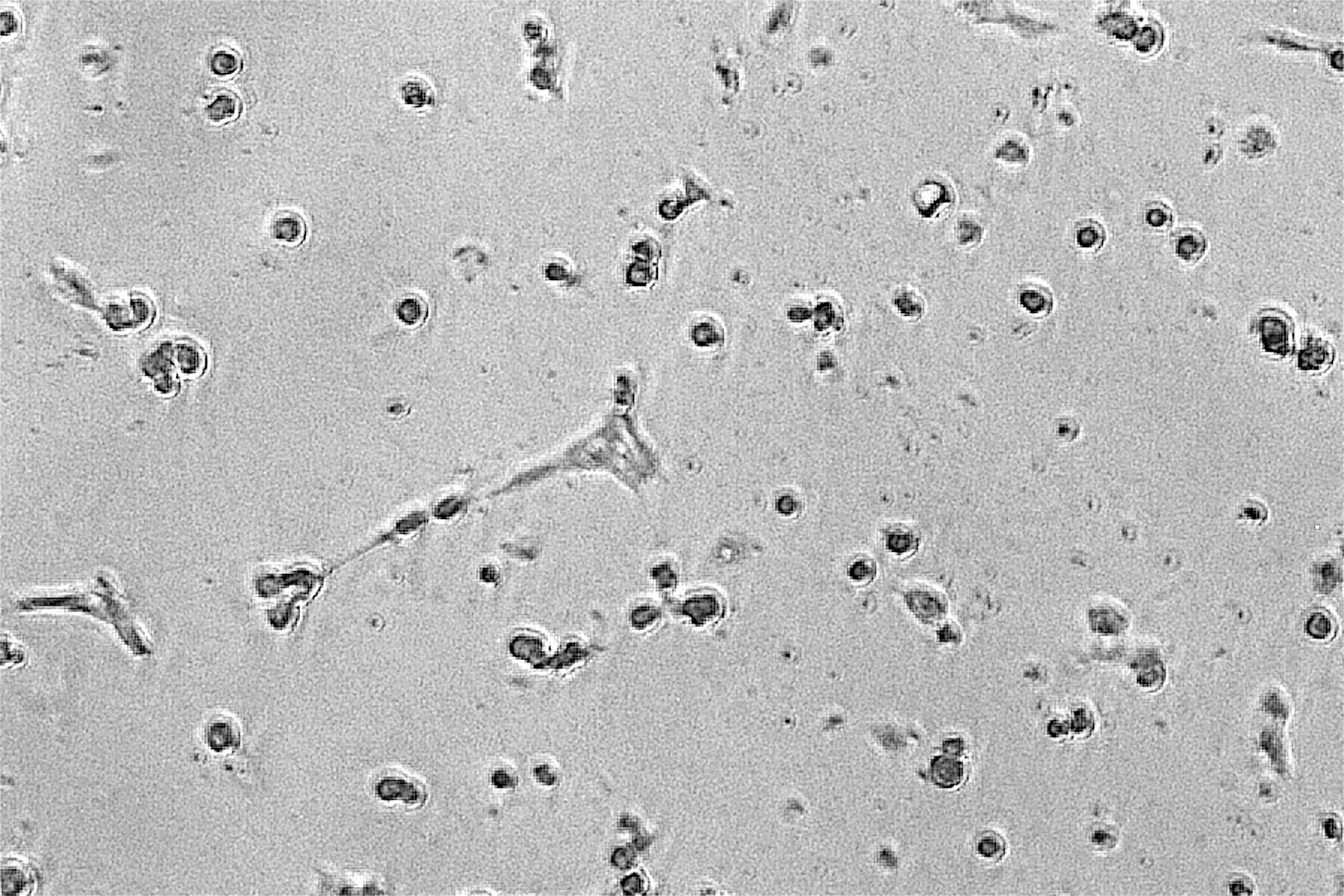

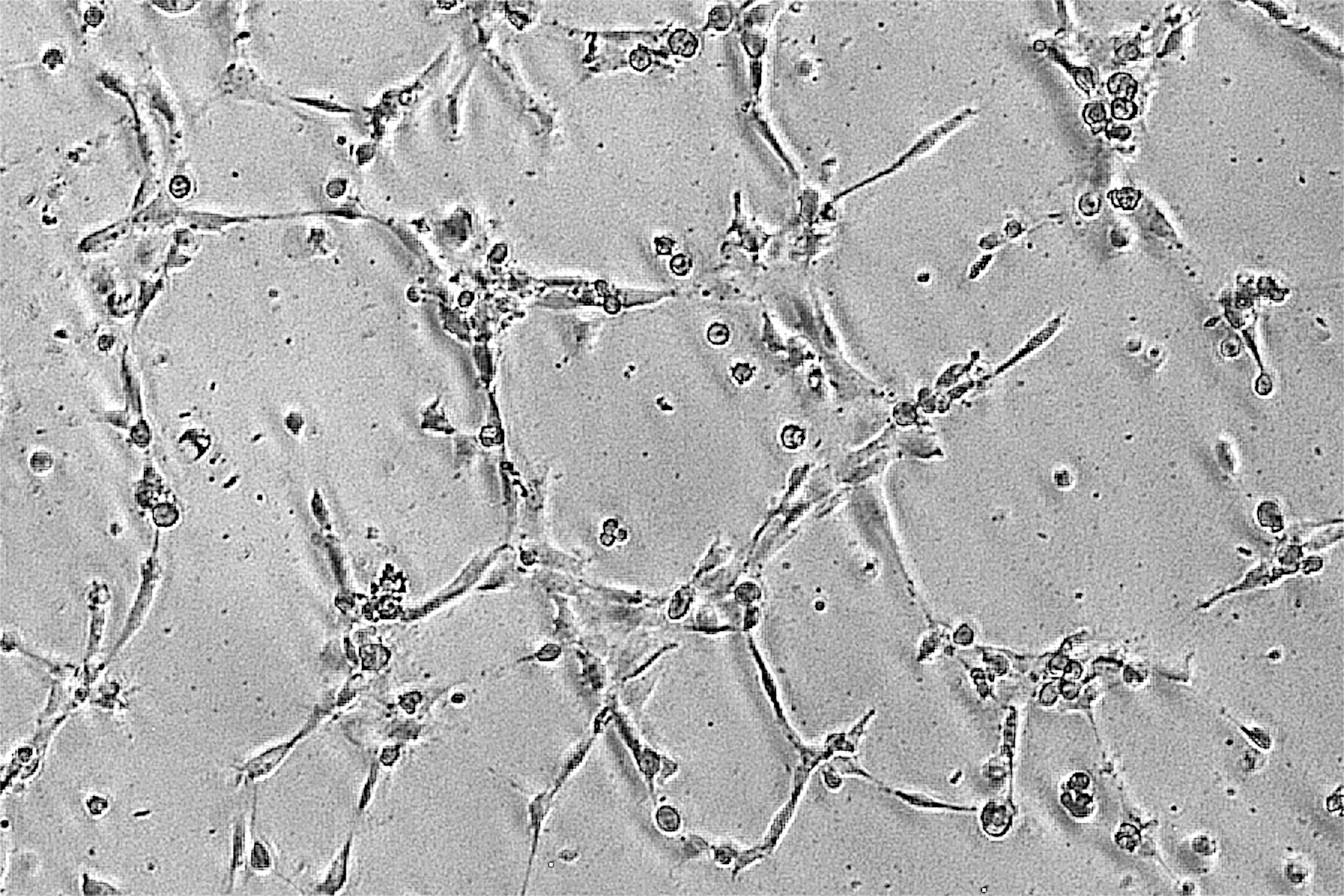

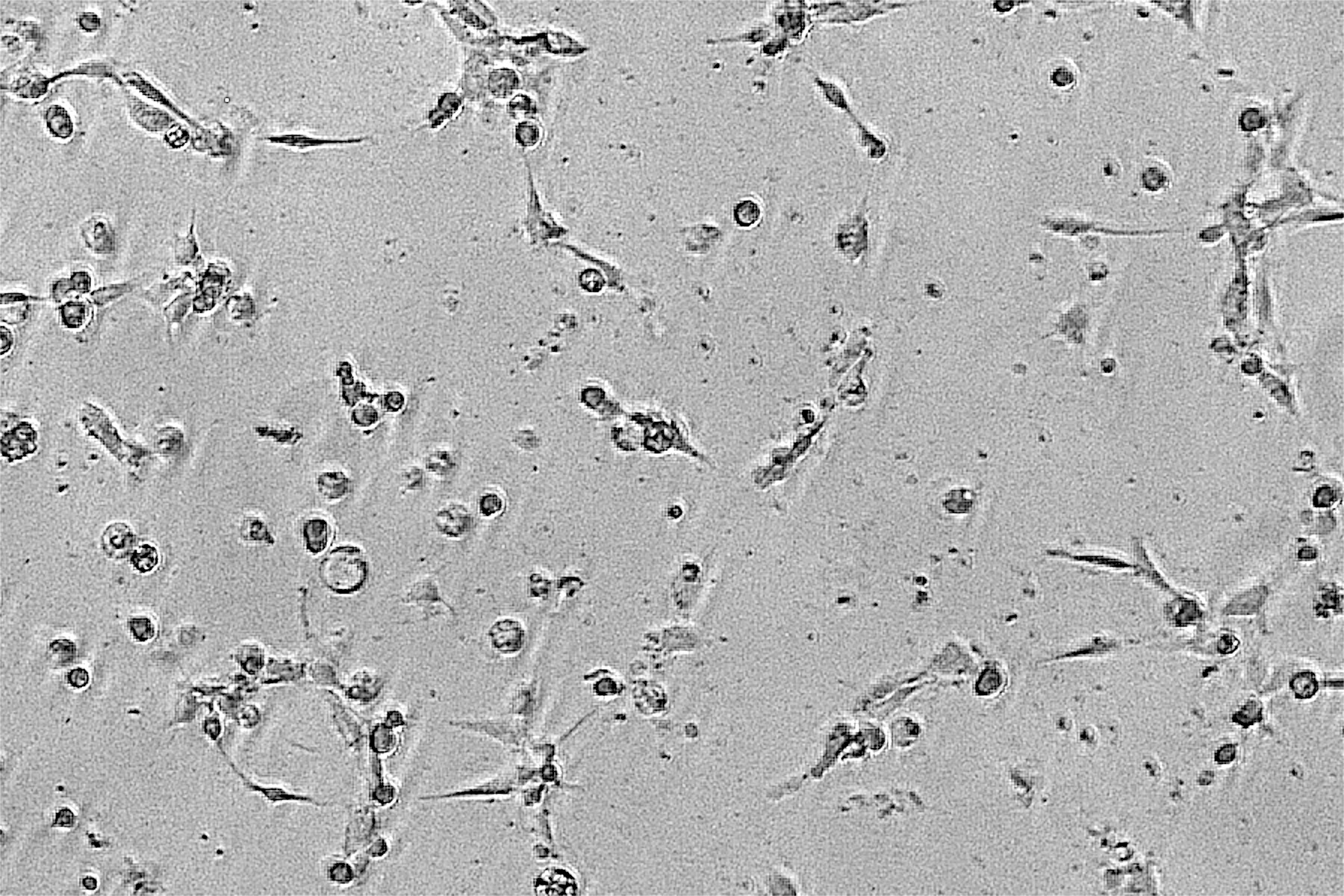

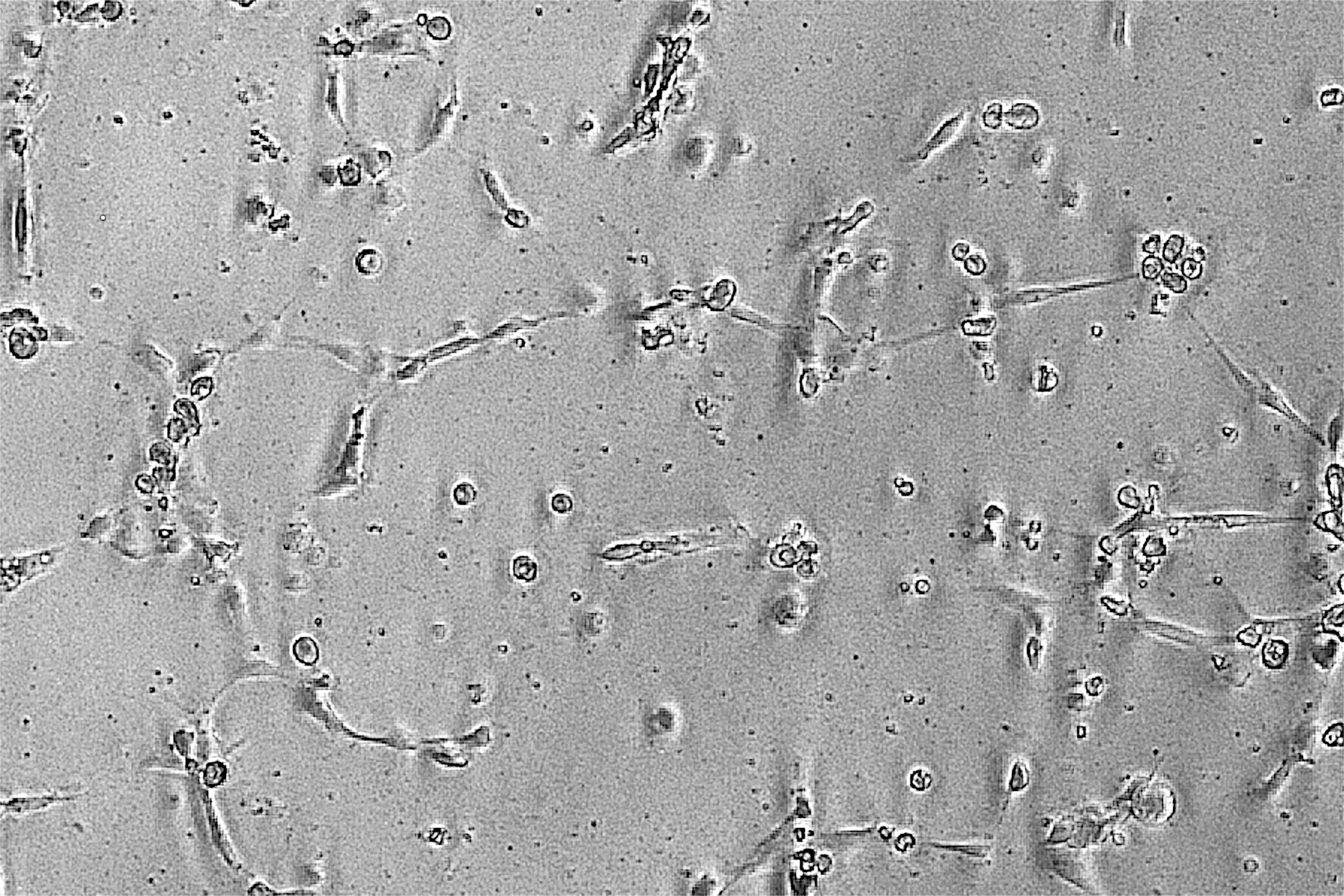


**A**

**B**

**C**

**D**

**E**

**Supplementary Figure S1.** **Neutralization of IL-17F and VEGF inhibits MyLa2059 supernatant-mediated endothelial tube formation**.

Endothelial tube formation assays were performed on growth factor reduced matrigel in 24 well plates. HUVEC cell sprouting when cultured with (A) M200 medium, (B) MyLa2059 supernatant (10% vol/vol), (C) MyLa2059 supernatant + anti-IL-17F antibody, and (D) MyLa2059 supernatant + anti-VEGF-A antibody (Avastin). (E) Quantification of number of branching points.


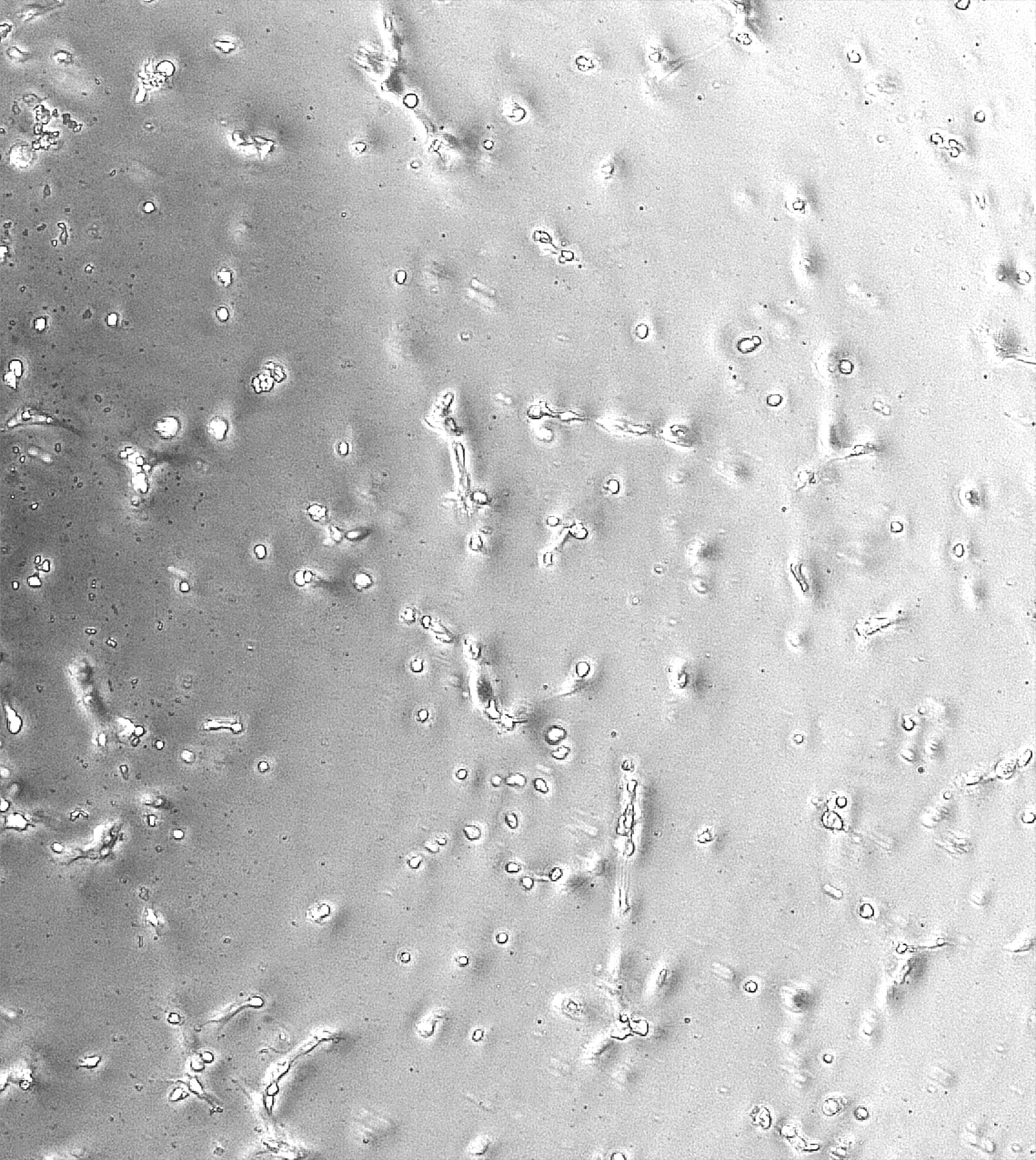

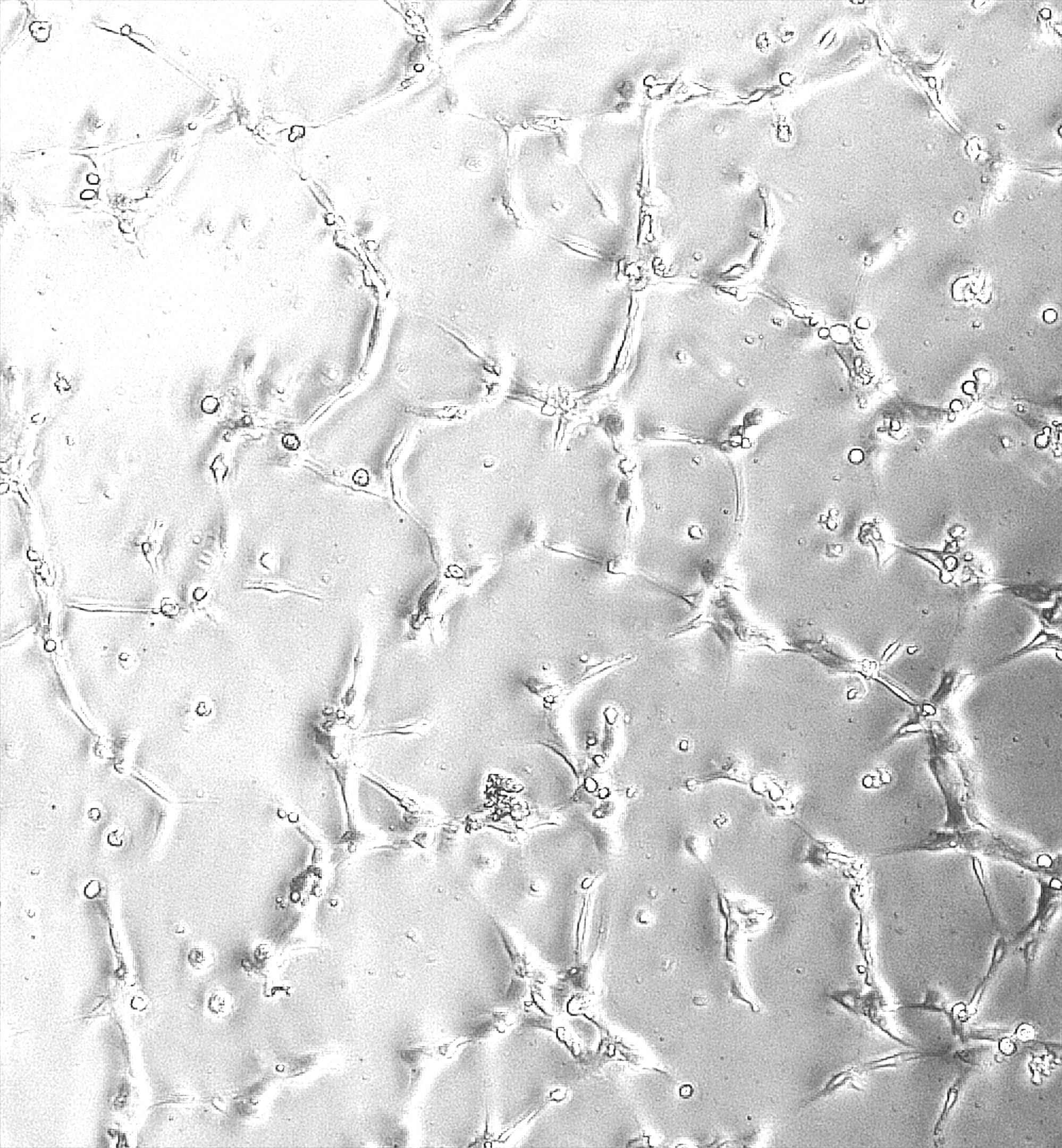

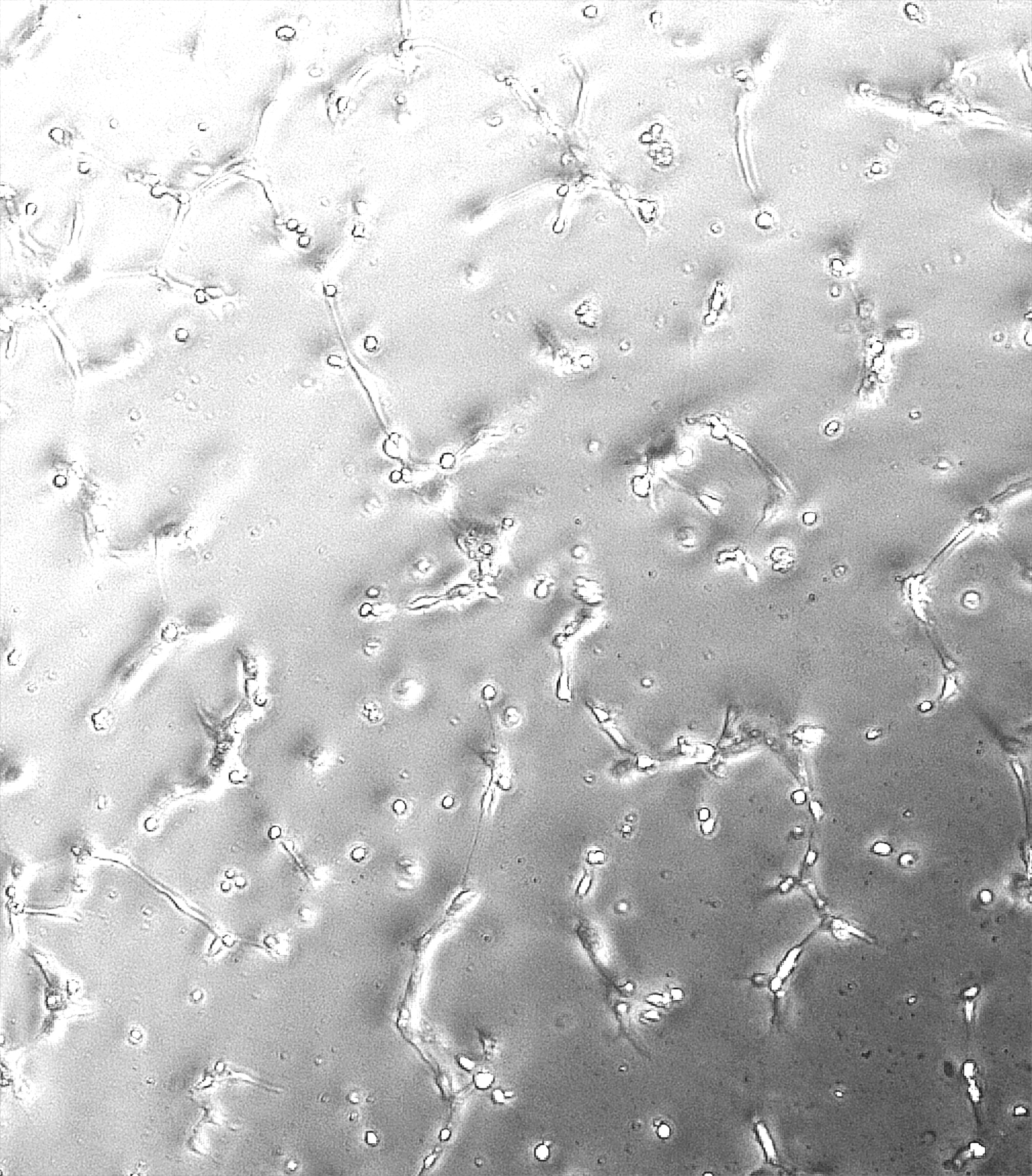


**A**

**B**

**C**

**Supplementary Figure S2. Malignant T cell line PB2B triggers IL-17F- mediated endothelial tube formation**.

Endothelial tube formation assays were performed on growth factor reduced matrigel in 24 well plates. HUVEC cell sprouting when cultured with (A) M200 medium, (B) PB2B supernatant (10% vol/vol), and (C) PB2B supernatant + anti-IL-17F antibody**.**

**
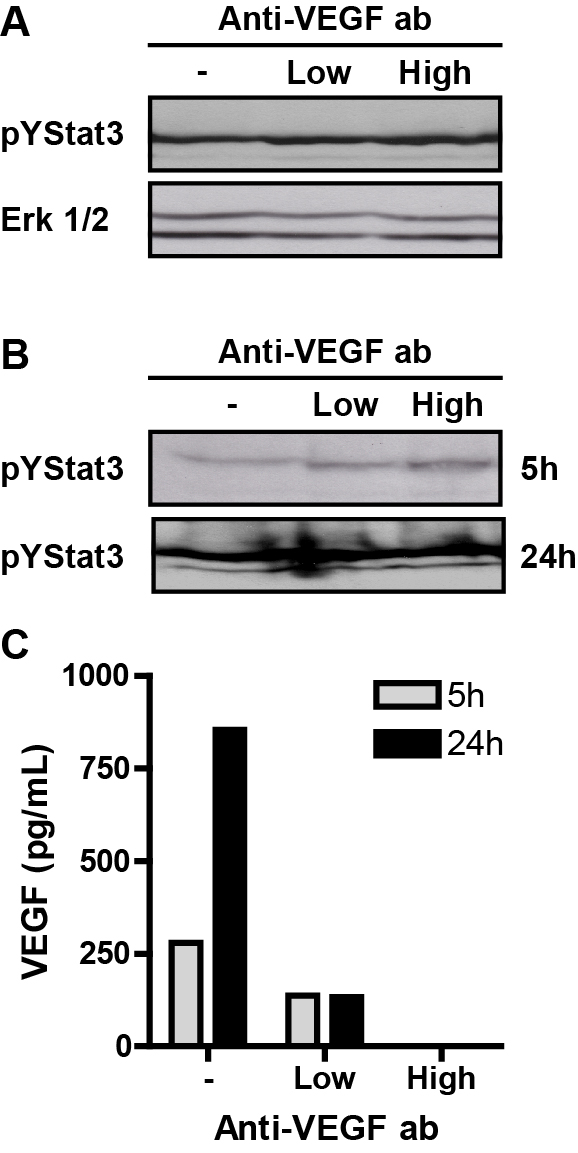
**

**Supplementary Figure S3. Autocrine VEGF signaling does not drive constitutive Stat3 activation in the malignant T cell line MyLa2059.** (A) Western blot showing the levels of phospho-Stat3 (pYStat3) and Erk1/2 in malignant T cells (MyLa2059) cultured for 24 hours in absence (-) or presence of low (20 ng/mL) and high (200 ng/mL) concentrations of a VEGF neutralizing antibody (R&D systems, Minneapolis, MN). (B+C) MyLa2059 cells were cultured in absence (-) or presence of low (100 ng/mL) and high (1000 ng/mL) concentrations of a VEGF neutralizing antibody (Leinco Technologies, St. Louis, MO) for 5 and 24 hours. (B) The cellular levels of pYStat3 were subsequently analyzed by western blotting while (C) the neutralizing antibody was depleted from the cell culture supernatants and the concentration of unbound VEGF determined by ELISA.
